# Effectiveness and cost-effectiveness of a group-based intervention to improve social-emotional development of young children in poverty-stricken areas: A cluster randomized controlled trial

**DOI:** 10.7189/jogh.13.04017

**Published:** 2023-02-03

**Authors:** Mengxue Xu, Haijun Zhang, Aihua Liu, Chunxia Zhao, Xiaona Huang, Stephen Berman, Hai Fang, Hongyan Guan

**Affiliations:** 1 Nurturing Care Research and Guidance Center, Capital Institute of Pediatrics, Beijing, China; 2 China Center for Health Development Studies, Peking University, Beijing, China; 3 Department of Health Policy and Management, School of Public Health, Peking University, Beijing, China; 4 Department of Integrated Early Childhood Development, Capital Institute of Pediatrics, Beijing, China; 5 Child Development Research Centre, China Development Research Foundation; 6 Child Health and Development, UNICEF China, Beijing, China; 7 Center for Global Health (CGH), University of Colorado Anschutz Medical Campus Aurora, Colorado, USA; 8 WHO Collaborating Center to Promote Family and Child Health, University of Colorado Anschutz Medical Campus Aurora, Colorado, USA; 9 Colorado School of Public Health, University of Colorado Anschutz Medical Campus Aurora, Colorado, USA; 10 Peking University Health Science Center – Chinese Center for Disease Control and Prevention Joint Research Center for Vaccine Economics, Beijing, China; 11 Institute for Global Health and Development, Peking University, Beijing, China

## Abstract

**Background:**

Social-emotional ability is key to the well-being and future success of children; however, disparities in social-emotional development during an individual’s early age can last a lifetime, which is particularly evident among children living in poverty-stricken areas. We aimed to determine the effectiveness, cost-effectiveness, and feasibility of a group-based intervention called the Care Group on social-emotional development for families living in poverty-stricken counties.

**Methods:**

We conducted a cluster (township) randomized controlled trial (C-RCT) every two weeks from July 2019 to June 2020 in a poverty-stricken area located in Shanxi, China. The outbreak of the COVID-19 pandemic suspended the implementation of the intervention in January 2020. The caregiver-child pairs in the intervention group participated in 12 group-based sessions with a structured curriculum and learning materials emphasizing nurturing ability and early childhood development. We applied a difference-in-differences (DID) model to estimate the intervention’s impact. The analysis follows the intention-to-treat (ITT) principle. We used standard economic costing methods to estimate the cost of implementing the Care Group over the intervention period and adopted a societal perspective in the analysis.

**Results:**

We included 322 eligible caregiver-child pairs in the baseline (intervention n = 136, control n = 186) and surveyed 258 pairs in the endline (intervention n = 117, control n = 141). Compared with the control group, children in the intervention group had significantly fewer social-emotional problems (adjusted mean difference of Z score = -0.374, 95% CI = -0.718, -0.030, *P* = 0.033) six months after intervention. In the first year, the annual cost of implementing Care Group was US$146.10 per child, reduced to US$47.20 per child in the second year due to the exclusion of non-recurrent costs. The incremental cost-effectiveness ratio (ICER) was US$390.60.

**Conclusions:**

Care Group is an effective approach for promoting children’s social-emotional development in poverty-stricken areas at an affordable cost and with high feasibility for scale-up. Considering the planned per capita health expenditure of the Chinese government for 2022, we believe that the presented evidence makes a solid scientific and financial case for integrating the Care Group intervention into the basic public health services (BPHS) package.

**Registration:**

Chinese Clinical Trials Registry (ChiCTR): ChiCTR1900022894.

There is increasing evidence confirming that social-emotional skills serve as the critical foundation for the well-being and future success of children; however, disparities in social-emotional development in the early years can last a lifetime, particularly evident among children living in poverty [1-3]. Previous studies found a prevalence of social-emotional delay ranging from 14% to 59%, with a weighted average of 36.5% among infants and toddlers in rural areas of China [4], while the prevalence in the entire Chinese sample was 20% [5]. An empirical study also found that the key window of opportunity for improving a child’s development is during or prior to the infancy stage [6]. Moreover, nurturing care practices have been positively correlated with children’s social and emotional development [7,8].

Group-based interventions have the advantages of modeling positive nurturing care behaviors and environments [9] and fostering a strong sense of belonging [10] at a lower cost [11]. Smaller group size (2-10 caregiver-child pairs), structured curricula, opportunities for practice among caregivers, feedback on nurturing care behaviors, and adequate intervention frequency were found to be crucial for successful group-based interventions [12]. Based on data from previous studies, the cost of group-based intervention on early childhood development (ECD) was about US$400-933 per child annually in the United States [13] and US$38-135 per child annually without non-recurrent costs in India [14].

An estimated 17 million children under the age of five in China were denied the opportunity to develop to their fullest potential due to various risk factors in 2017, such as child poverty, malnutrition, lack of psychosocial stimulation, and poor parenting environment [15]. While China has achieved remarkable progress in poverty alleviation [16], the incidence of early childhood developmental delays, especially for on non-cognitive development [17], has been worse [18,19] in poverty-stricken areas due to persistent wealth disparities between urban and rural areas [20].

We aimed to examine the effectiveness of the Care Group intervention on social-emotional development of children under the age of three, evaluate its cost-effectiveness, and determine the feasibility of the delivery paths (eg, facilitator selection, training, supervision) and compliance to its implementation in poverty-stricken areas in China. The data presented is not only relevant for expanding ECD services for children aged 0-3 in poverty-stricken areas of China, but also valuable to other countries with similar social and economic situation.

## METHODS

### Study design and participants

We conducted a cluster randomized controlled trial (C-RCT) every two weeks from July to December 2019 in eight townships of Fenxi County, a poverty-stricken area in Shanxi Province of China [21]. Gross domestic product (GDP) per capita in Fenxi County in 2019 was US$2151 [22], much lower than that of China (US$10 144) [23] and Shanxi Province (US$2465) [24] in 2019. We conducted the endline evaluation survey six months later in July 2020 due to the interruption of the COVID-19 pandemic.

The intervention group participated in Care Group Intervention while also receiving child health care services included in the basic public health services (BPHS) package, while the control group received only the child health care services included in the BPHS package. We intended for the caregiver-child pairs in the intervention group to participate in 24 group-based sessions over 12 months between July 2019 to June 2020. However, due to the COVID-19 pandemic, the pairs in the intervention group were only able to participate in a maximum of 12 group-based sessions from July to December 2019. Since January 2020, we suspended the offline intervention for six months, and facilitators switched to sending age-appropriate messages and relative links of nurturing care and child health to caregivers via Wechat (a free mobile application for text and voice messaging communication service), to encourage them to keep practicing and maintain positive nurturing practices learned before.

Recruited by the village doctors, children who were aged 6-18 months by July 1, 2019, and their primary caregivers were eligible for inclusion in the intervention group. Each primary caregiver signed the informed consent form after confirming they completely understood the content and information. Exclusion criteria for data analysis were: 1) children with physical disabilities; 2) multiple births (eg, twins, triplets); 3) premature babies; and 4) children living more than five kilometres from the intervention site.

### Sample size and power calculation

The Care Group Intervention is designed to increase children’s social-emotional development by 0.35 standard deviations (SDs). Assuming a 95% confidence interval (CI) and a power of 0.80, we calculated the minimum sample size as 102 per group using the G*Power 3.1 software. We estimated a 90% valid data and 80% follow-up rate. We thus aimed to recruit 142 participants in each group, with a total 284 participants (142 × 2).

### Randomization and masking

We matched the townships (n = 8) into pairs based on the order of the socioeconomic index (per capita disposable income in 2018). Each township was marked as A1, A2, B1, B2, C1, C2, D1, and D2 after four pairs were decided. We used IBM SPSS V.24.0 (IBM Corp) to randomly choose one number in each pair as the intervention township, after which its paired township automatically became a control group. We assigned all the children living in intervention/control townships to the intervention/control groups. We could not blind the participants and facilitators due to the nature of the Care Group intervention.

### Care Group intervention

The Care Group Manual, developed by the University of Colorado in the United States, has been proven as a practical tool to improve the health and development of children aged 0-3, and advance caregivers’ knowledge and skills on nurturing care in Guatemala [25,26] Adapted to the Chinese context, the Care Group Flipchart and the Care Group Toolkit were developed as part of the ECD program (2017-2020) jointly supported by UNICEF and the National Health Commission of China.

The Care Group intervention aims to promote children’s comprehensive development by strengthening primary caregivers’ knowledge and skills on nurturing care practices. The focus of the Care Group content spans five key components (good health, adequate nutrition, responsive caregiving, security and safety, and opportunities for early learning) under the Nurturing Care Framework promoted by the World Health Organization and UNICEF [27]. We emphasized the five fundamental philosophies (responsive caregiving, sufficient early stimulation, play and communication, encouragement and praise, and security) and the corresponding nurturing care skills during the entire intervention. After brief lectures on child health and nurturing care at the beginning of each session, two facilitators demonstrated nurturing care skills, organized play/reading interaction activities, and responded to the parents’ and caregivers’ questions. Each session lasted 45 minutes and had a structured curriculum. The agenda is available in Figure S1 in the **Online Supplementary Document**. Facilitators recorded participation, children and caregiver performance, and questions/feedback after each session. More details on the Care Group can be found in the published protocol [21].

### Delivery path

We delivered the Care Group intervention through the BPHS system at the township level. We established a three-tiered supervision mechanism at the national, county and village levels, carrying out quality assurance at the national and county levels at a quarterly, and at the village level at a monthly basis. We selected nine local women (including one health worker, one family planning worker, three experienced volunteers, three female caregivers, and one kindergarten teacher) who at least completed senior secondary education as facilitators after rounds of interviews and training. We used a two-phase training to help facilitators familiarize with the key messages and skills outlined in the Care Group Manual, and the training specifically emphasized and demonstrated how to promote positive relationships and cultivate a warm environment for participants.

### Measurement and data analysis

We evaluated the children’s social-emotional development using the Ages & Stages Questionnaires: Social Emotional, Second Edition (ASQ: SE-2), a caregiver-reported screening tool to identify social-emotional problems among children aged 1-72 months; The ASQ: SE-2 has been introduced in many countries and is widely used in research and clinical settings. It has been adapted to the Chinese context and approved as a reliable tool for assessing the social-emotional development of children aged 0-6 [25]. The tool includes 16 to 36 questions depending on the specified age group, and caregivers are asked to provide answers on a three-point Likert scale. The questions focus on typical behaviors for the specified age group in seven dimensions, including self-regulation, compliance, communication, adaptive functioning, autonomy, affect, and interaction with others. The total score is the sum of each question, and a higher score indicates poorer social-emotional development.

We trained four graduate students to help caregivers log into the ASQ: SE-2 electronic system which provides uniform illustration and audio-visual materials. Raw data were automatically uploaded. Child demographic characteristics (including gender, age and order of birth) and maternal demographic characteristics (including education, marital status, attendance, caregiver’s satisfaction toward intervention and socioeconomic status) were chosen as control variables. All questionnaires were administered by the GoodData software (GoodData, Beijing, China) using an Android system. We set the range and logistics of each response in advance to guarantee data accuracy completeness.

According to the rules of the intention-to-treat (ITT) analysis, we performed the data distribution before the data analysis. For the descriptive analysis on the children (age group, gender, and birth order), parents (i.e. education, age, and stay-at-home parent) and the family’s socioeconomic status (i.e. annual income in 2018, vehicle ownership, and household appliance ownership), we used *t* test or χ^2^ test. We applied a difference-in-differences (DID) model to estimate the intervention’s impact on the children’s social-emotional developmental outcomes. We calculated a Z-score according to each child’s score on ASQ: SE-2 in their age group. We reported the odds ratios (ORs), 95% confidence intervals (CIs), coefficient of control variables, and their *P*-values, setting the threshold for statistical significance at *P* > 0.05 (bilateral). We conducted the data analysis using IBM SPSS Statistics 24.0.

### Cost-effectiveness analysis

We used standard economic costing methods to estimate the cost of implementing the Care Group over the intervention period [28] and adopted a societal perspective in the analysis adopted. We included all intervention-related costs: recruitment, initial training, group material (e.g. basic parenting pack, books, dolls etc.), and care group costs. We collected all costs data through qualitative interviews with implementing staff and referenced the intervention budget and expenditure records. We utilized the human capital approach [29] to calculate the time cost of all staff. We converted all the calculated costs in 2019 from Chinese RMB to US dollars (US$1 = RMB6.908). We calculated incremental cost-effectiveness ratios by dividing the intervention costs by the differences between the intervention and control groups in terms of developmental outcomes (i.e. overall ASQ: SE-2 Z scores). As we obtained all costs within a one-year time horizon, discounting costs or effects were not needed. We used univariate sensitivity analyses to determine the impact of cost variation by allowing the personnel costs to fluctuate up and down by 50%.

## RESULTS

**Figure 1:**
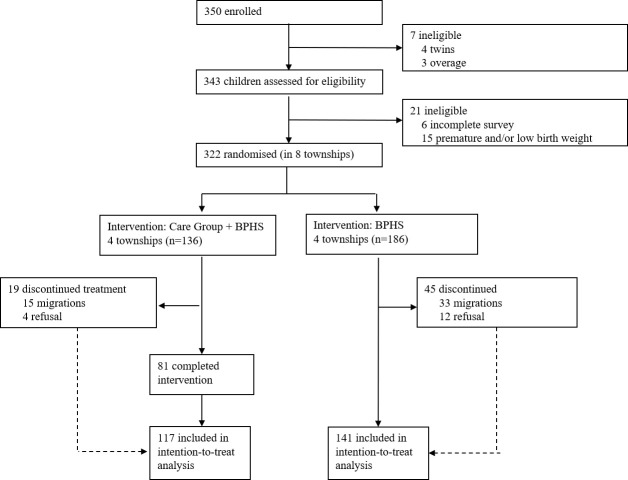
Trial profile; BPHS – basic public health service.

We recruited 322 children aged 6-18 months recruited in the baseline survey, assigning 136 to the intervention group and 186 to the control group. The endline survey in July 2020 included 117 children from the intervention group (86.03%) and 141 children from the control group (75.81%). Forty-eight caregiver-child pairs dropped from the project due to migration (intervention group n = 33; control group n = 15). The specific exit time and drop-out reasons from the intervention group are available in Table S1 in the **Online Supplementary Document**. Sixteen caregivers refused to participate in the endline survey (intervention group n = 4; control group n = 12) (**Figure 1****)**.

### Comparison of demographic characteristics between intervention and control groups

The demographic characteristics were normally distributed. At the baseline, there was no significant difference in the demographic characteristics of children (e.g. age group, gender, and birth order) between the intervention and control groups (**Table 1**). Mothers in the intervention group were significantly younger than those in the control group (intervention = 27.89; SD = 3.44; control = 29.20; SD = 4.13; *P* = 0.007). We found no significant difference in the mother’s level of education, father’s level of education, and age. The proportion of stay-at-home (not working) mothers was 81.2% in the intervention group and 85.5% in the control group, but there was no statistically significant difference (*P* = 0.317). Even though annual incomes (intervention = 5582, SD = 4824; control = 5334, SD = 3935; *P* = 0.685) in 2018 were similar in both groups, the quality of life in the control group was better than the intervention group (*P* < 0.05), as demonstrated by the ownership of vehicles (i.e. motorcycle and family car) and household appliances (i.e. air conditioner and tablet).

**Table 1:** Baseline characteristics of the intention-to-treat population

Child	Intervention group (n = 117), n (%)	Control group (n = 141), n (%)	*P*-value
**Age group (in months)**			0.517
6-11	62 (47.3%)	69 (52.7%)	
12-18	55 (43.3%)	72 (56.7%)	
**Gender**			0.407
Male	57 (48.7%)	76 (53.9%)	
Female	60 (51.3%)	65 (46.1%)	
**Birth order**			0.575
First	56 (47.9%)	55 (39.0%)	
Second	49 (41.9%)	66 (46.8%)	
Third or later	12 (10.2%)	20 (14.1%)	
**Mother**			
Education			0.591
*Primary school and below*	5 (4.3%)	10 (3.9%)	
Junior secondary school	54 (46.2%)	62 (44.0%)	
*Senior secondary school*	37 (31.6%)	44 (31.2%)	
*College and above*	44 (17.1%)	25 (9.7%)	
Age (in years), mean (SD)	27.89 (3.44)	29.20 (4.13)	0.007
Stay-at-home	95 (81.2%)	121 (85.8%)	0.317
**Father**			
Education			0.222
*Primary school and below*	3 (1.2%)	8 (3.1%)	
*Junior secondary school*	71 (60.7%)	70 (49.6%)	
*Senior secondary school*	28 (23.9%)	41 (29.1%)	
*College and above*	3 (1.2%)	4 (1.6%)	
Age (in years), mean (SD)	30.00 (4.04)	31.18 (7.06)	0.109
**Socioeconomic status**			
Annually income in 2018 (US$)*	5582 (4824)	5334 (3935)	0.685
Vehicle			
*Motorcycle*	95 (81.2%)	92 (65.2%)	0.004
*Motorized bicycle*	49 (41.9%)	51 (36.2%)	0.349
*Family car*	56 (47.9%)	87 (61.7%)	0.026
Household appliances			
*Air Conditioner*	15 (12.8%)	35 (24.8%)	0.015
*Computer*	50 (42.7%)	71 (50.4%)	0.222
*Tablet*	7 (6.0%)	23 (16.3%)	0.010

SD – standard deviation

*US$1 = RMB6.861, 2018 exchange rate from World Bank (https://data.worldbank.org/).

### Comparison of social-emotional changes between intervention and control groups

The Z-scores in the intervention group were slightly higher than in the control group at the baseline, without a significant difference (intervention = 0.099 SD = 1.040; control = -0.082; SD = 0.942; *P* > 0.05). In the endline survey, however, the Z-scores were significantly lower in the intervention group (intervention = -0.106, SD = 0.952; control = 0.088, SD = 1.026; *P* < 0.05). The alteration (i.e. intervention group minus control group) was significantly different (difference-in-difference = -0.374, *P* < 0.05), demonstrating that children in the intervention group had fewer social-emotional problems (95% CI = -0.718, -0.030, *P* < 0.05, **Table 2**). The variation tendency is presented in Table S2 in the **Online Supplementary Document**.

**Table 2:** Social-emotional changes in the intervention and control groups (Z-scores)*

	Mean (SD)		Difference-in-difference (intervention – control)	95% CI	SE	t-value	*P*-value
	**Intervention**	**Control**					
Baseline	0.099 (1.040)	-0.082 (0.942)	0.181	N/A	0.128	0.840	0.401
Endline	-0.106 (0.952)	0.088 (1.026)	-0.194	N/A	0.128	-2.091	0.037
	Difference-in-difference		-0.374	-0.718, -0.030	0.175	-2.139	0.033

SD – standard deviation, CI – confidence interval, N/A – not applicable

*A lower Z-score indicates fewer social-emotional problems. There was no significant difference of the Z-scores in the baseline (*P* = 0.144) and endline (*P* = 0.121) surveys. Maternal age, gender of child, socioeconomic index (e.g. ownership of motorcycle, family car, air conditioner, tablet), and participation rate were adjusted in the difference-in-difference analysis.

### Total cost and cost per child of implementing group-based intervention

**Table 3** summarizes the cost of implementing the group-based intervention. The cost of implementing twelve sessions per child was US$146.10. The costs for recruitment, training, materials, and monthly operation of Care Group were US$558.90, US$11 011.70, US$2686, and US$2834.20, respectively, with the training costs accounting for the highest proportion. When we excluded non-recurrent costs (i.e. recruitment and training costs), the cost per child was US$47.20. Details of collection of cost items are shown Tables S3-1 to S3-4 in the **Online Supplementary Document**.

**Table 3:** Total cost and cost per child of running the group-based intervention (in 2019, USD)*

Cost item	Unit	Unit cost	Total cost†
**Recruitment costs**			
Cost for recruiting parenting facilitators	Nine parenting facilitators (41.5 min per person)	27.30 per person	245.70
Cost for recruiting parenting supervisors	Four supervisors (41.5 min per person)	27.30 per person	109.20
Cost for recruiting caregivers	136 caregivers (intervention group) (10 min per person)	1.50 per person	204
**Subtotal**			**558.90**
Initial training costs			
*Time for five training experts*	Nine days (eight hours per day)	122.40 per day	5508
*Time for nine parenting facilitators*	Nine days (eight hours per day)	7.20 per day	583.20
*Time for four parenting supervisors*	Nine days (eight hours per day)	12 per day	432
*Transportation fee*	Nine days	16.10 per day	144.90
*Accommodation fee*	Nine days	234.50 per day	2110.60
*Food and catering*	Nine days	156.30 per day	1407.10
*Other costs (e.g. office expenses)*	One time	825.90 per program	825.90
**Subtotal**			**11 011.70**
Material costs (e.g. basic parenting pack, books, dolls etc.)	Nine facilitators	298.40 per facilitator	2686
Monthly Care Group costs			
*Monthly supervision cost for one local women’s federation staff*	Six times per program	118.30 per time	709.80
*Cost for nine parenting facilitators*	Seven times per person per month (45 min per time)	3.70 per person per month	1398.60
*Costs for four parenting supervisors*	5.6 times per person per month (54.2 min per time)	5.40 per person per month	725.80
**Subtotal**			**2834.20**
**Cost of implementing group-based intervention**			
Total cost including non-recurrent costs (recruitment and training costs)			17 090.80
Cost per child (if children per group)			146.10
Total cost excluding non-recurrent costs			5520.20
Cost per child (if children per group) excluding non-recurrent costs			47.20

*US$1 = RMB6.861, 2018 exchange rate from World Bank (https://data.worldbank.org/).

†In some cases, the total costs do not equal to the multiplication of the unit and the unit cost because of rounding.

### Incremental cost-effectiveness ratios and univariate sensitivity analysis

The incremental cost-effectiveness ratios (ICERs) with and without non-recurrent costs were US$390.60 and US$126.20, respectively. The univariate sensitivity analysis showed that the up-and-down variance of the personnel costs by 50% yielded intervention costs per child of US$103.70 to US$188.50, with corresponding ICERs varying from US$277.30 to US$504.00. When non-recurrent fees were excluded, the cost per child ranged from US$35.10 to US$59.30, and the ICERs varied from US$93.90 to US$158.60 (**Table 4**).

**Table 4:** Incremental cost-effectiveness ratios and univariate sensitivity analysis by varying the personnel costs (in USD)

Variation of personnel costs*	Cost per child		ICER (overall ASQ: SE-2)	
	Including non-recurrent costs	Excluding non-recurrent costs	Including non-recurrent costs	Excluding non-recurrent costs
Base case	146.10	47.20	390.60	126.20
-50%	103.70	35.10	277.30	93.90
+50%	188.50	59.30	504.00	158.60

ICER – incremental cost-effectiveness ratio, ASQ: SE-2 – Chinese version of Ages and Stages Questionnaires: Social-Emotional, second edition

*Includes recruitment costs, initial training costs for experts, parenting facilitators and supervisors, and care group

## DISCUSSION

To the best of our knowledge, this is the first study in China to evaluate the effectiveness and cost-effectiveness of group-based intervention on the social-emotional development of children in poverty-stricken areas. The positive findings indicated that the Care Group is scalable. Most importantly, the endline survey found that the Care Group had a sustained positive impact on the social-emotional development of children six months after the intervention was interrupted by the outbreak of the COVID-19 pandemic.

### Effectiveness of the Care Group on social-emotional development

The positive effect of group-based intervention on promoting social-emotional development in our findings was similar to or larger than the effect found by previous studies [4,9]. Hamadani et al. [9] had a much larger effect size in Bangladesh because the intervention targeted underweight children and lasted longer. Based on our analysis, there are three key factors that contributed to this positive effect. First, a safe and secure environment is fundamental for children to explore their surroundings and interact with others [30]. The Care Group provided children with this opportunity and encouraged caregivers to extend positive surroundings full of stimulate and nurturing care practices at home. Second, modeling played an essential role. According to social learning theory [31], children and caregivers tend to learn from observations through imitation and internalization. In our study, we focused on improving the nurturing care behaviors through lectures and discussions, demonstrations of responsive care practices, and effective feedback for caregivers. We also showed that the positive nurturing behaviors in the intervention group significantly increased after the intervention (Table S4 in the **Online Supplementary Document**). One study [32] showed that the link between parenting skills and the social-emotional development of children was more vital than the cognitive, language, and motor development of children. Third, the Care Group provided a valuable opportunity for caregivers to openly share their feelings and thoughts. Depressive symptoms, parenting stress, and marital adjustment have been significantly associated with maternal sensitivity [33-35] and a mother’s ability to promptly and appropriately respond to their infant’s behavioral signals. We found that mothers in the intervention group received more peer support, and they were more willing to share their inner thoughts and feelings after a few sessions.

### Cost-effectiveness of the Care Group

Several studies targeting group-based intervention on ECD were found to be cost-effective and efficient in other countries [36-39]. For example, the Video Interaction Project [40], a targeted intervention in the pediatric primary care setting designed to enhance parenting skills and boost school readiness in the United States, has an annual cost of US$150-200 per child. The Incredible Years [41] parenting program in the United Kingdom, with costs ranging from £1612-2418 (US$2524-3787) per child depending on the number of children in the group, was found to have a high probability of being cost-effective. The cost of our group-based intervention adjusted by consumer price indices, ranging from US$50-150, is relatively lower and more efficient compared with these programs. Compared with other domestic studies in China, such as the one by Shi et al. [42] on parenting intervention and ECD, the average costs are very similar, yet we believe the Care Group may be more cost-effective because we implemented our intervention in rural areas where children and caregivers have decreased access to resources and services. Moreover, if the number of children in each Care Group doubles from four to eight children per group, the average cost of the project will be further reduced, greatly increasing the return on investment.

From the perspective of government health investment, the public health expenditure per person was US$0.32 (RMB2.20), calculated by dividing the total cost of the Care Group by the population of Fenxi County (104 627 permanent residents). According to the governmental project report of Basic Public Health Service in 2021 [43], Chinese government would increase the per capita public health expenditure by RMB5 (US$0.72) beginning in 2022. Therefore, available government funds can fully cover the total costs of the Care Group.

### Feasibility of scaling up the Care Group nationwide

We found the implementation of the Care Group to be feasible regarding the delivery path, facilitator selection, capacity building, and materials preparation. The three-tiered “national-county-village” supervision mechanism relied on the existing health system and guaranteed the quality of the implementation of the Care Group. This supervision mechanism provided an excellent preliminary foundation for sample enrolment, facilitator recruitment, training, and regular supervision.

While selecting potential facilitators, we prioritized a candidate’s capacity for empathy, their level of responsibility and communication, and relevant work experience. With the continuous improvement of education access among women and the growing awareness of the importance of ECD in rural areas of China, many full-time mothers of children under the age of three have been great candidates as facilitators. We also ensured sufficient pre-intervention training and frequent capacity building. According to the feedback from supervisors, the two-week training before implementation of the intervention and the high frequency of trainings in the first three months of implementation played an essential role in developing and improving the skills of supervisors and facilitators.

Finally, the Care Group Toolkit materials were economical, portable, and substitutable (see Table S5 in the **Online Supplementary Document**), allowing caregivers the possibility to purchase or make similar toys. This was beneficial for the creation of home-based early learning opportunities. We believe that our findings demonstrate the scalability of the Care Group intervention to a national level and its potential for integration the Care Group into the BPHC package.

### Limitation

Our study had some limitations. The increasingly apparent circumstance of frequent migration between the townships and the nearby urban areas impacted both enrolment and participation. With better educational and medical resources, job opportunities, and recreational facilities, young parents tend to work in nearby counties or urban areas. Meanwhile, they choose to settle down in the townships because of the low level of consumption [44]. Another limitation was the interruption of the Care Group intervention due to the COVID-19 pandemic. We could neither implement the intervention on-site nor adequately assess the immediate outcome and the social-emotional development of children. To adapt to the COVID-19 context, local facilitators used WeChat to maintain limited online interaction with caregivers. While we did not survey and quantify the level that the caregivers put into effect, this might be a reason of the sustained effect. This is also an interference factor for cost-effectiveness. Because the network system in China is very convenient and inexpensive, we had no expense for training and supervision in message transferring; we assumed no economic input was included during the epidemic period. The effect immediately after intervention was supposed to be greater than it was six months later, so we still believe the current results to be conservative and credible.

### Further study

During the implementation, we were concerned with the overall participation rate (**Online Supplementary Document**). Lack of awareness and attention on ECD were also related to some caregivers’ absence. Strategies to alleviate these difficulties should be considered in future studies. Moreover, researchers can further explore diversified forms of intervention. For example, a mixed media approach may be more engaging and interactive for caregivers and children; intervention frequency can be altered to examine the most appropriate frequency; and caregiver-only sessions can be implemented to assist in deeper learning of nurturing care knowledge and sharing of feelings and challenges with less distraction.

## CONCLUSIONS

The Care Group is an effective approach for promoting social-emotional development of young children in poverty-stricken areas at an affordable cost and with high feasibility for scale-up. Considering the planned per capita health expenditure of the Government of China for 2022, we believe that the evidence presented makes a solid scientific and financial case for integrating the Care Group intervention into the BPHS package.

## Additional material


Online Supplementary Document
