# Supplementary material for: Effectiveness and cost-effectiveness of a group-based intervention to improve social-emotional development of young children in poverty-stricken areas: A cluster randomized controlled trial
Source: J Glob Health. 2023 Feb 3;13:04017. doi: 10.7189/jogh.13.04017 (PMC9896863; doi:10.7189/jogh.13.04017)
Supplement: Online Supplementary Document [file jogh-13-04017-s001.pdf]

ONLINE SUPPLEMENTARY DOCUMENT

Title: Effectiveness and cost-effectiveness of a group-based intervention to improve social-emotional development for young children in poverty-stricken areas: a Cluster RCT

Authors: Mengxue Xu, Haijun Zhang, Aihua Liu, Chunxia Zhao, Xiaona Huang, Stephen Berman, Hai Fang, Hongyan Guan

Online Supplementary Document 1: Care Group Agenda

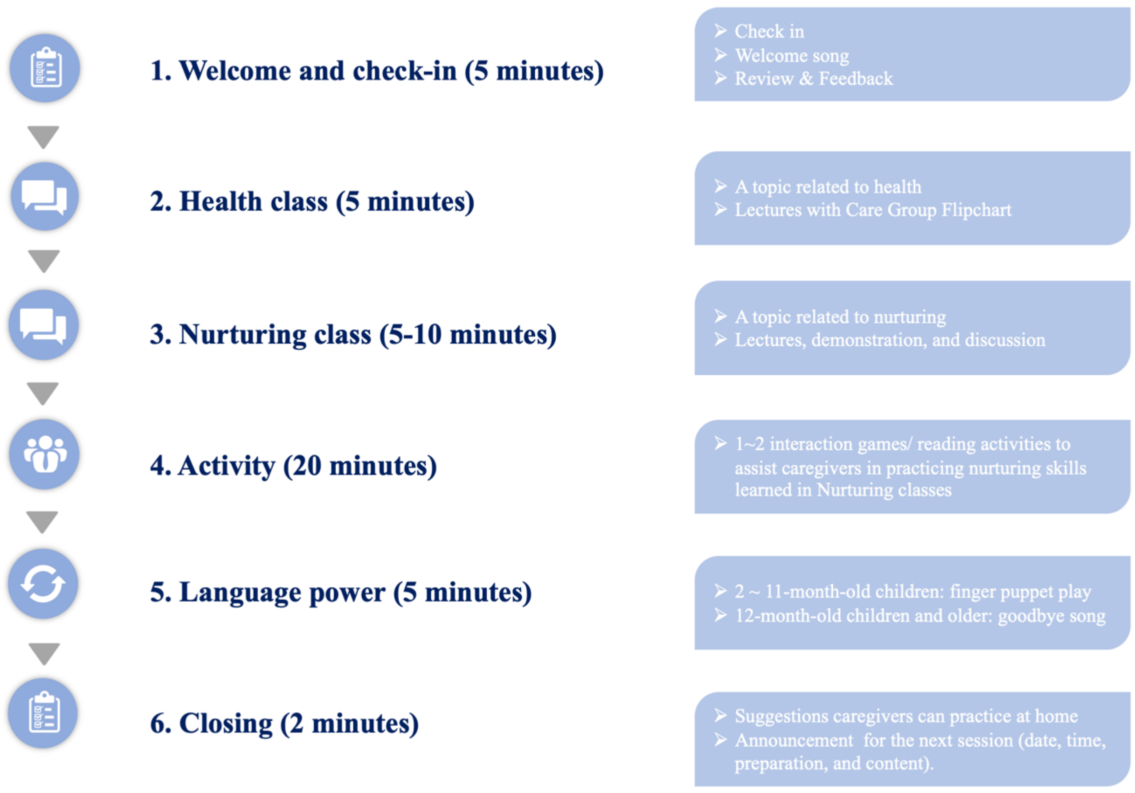

Figure S1 Gare Group agenda

Online Supplementary Document 2: Compliance of the intervention group

We classified the caregiver-child pairs in the intervention group into four groups according to their participation frequency: Excellent participation (n=10-12 sessions), Good participation (n= 6-9 sessions), Acceptable participation (n=3-5 sessions), Minimum participation (n= 1-2 sessions), No participation (n=0). Caregiver-child pairs with Good to Excellent participation were supposed to have a significant effect in ECD and nurturing care practice compared with control group, while those with acceptable participation and above were supposed to have changes in children's social-emotional development and nurturing care practices.

The percentage of Excellent, Good, Acceptable, Minimum, and No participation in the Care Group intervention was 16.24%, 17.95%, 26.50%, 8.55%, and 30.77%, respectively. The cumulative percentage of Acceptable participation and above was 60.68% (Appendix 4). For the caregiver-child pairs with Acceptable or Minimum participation, the majority (n=29) of them dropped from the intervention during July to August 2019, 1 or 2 months after the initiation of the project. Among them, 26 children migrated to a nearby county due to their elder brother/sister's entering school in September (21 children) or children's parents working in other counties (5 children); the other 3 caregivers refused to tell the reason in the endline survey. Caregiver-child with Good participation reported multiple reasons for absence such as long-distance, difficulty in transportation, and family illness.

Table S1 Compliance of the intervention group

Standard (n)	No. of Participation	Percentage	Cumulative percentage
Excellent participation (n=10-12)	19	16.24%	16.24%
Good participation (n= 6-9)	21	17.95%	34.19%
Acceptable participation (n=3-5)	31	26.50%	60.68%
Minimum participation (n= 1-2)	10	8.55%	69.23%
No participation (n=0)	36	30.77%	100%

Online Supplementary Document 3: Prevalence of social-emotional delay among intervention and control groups

The social-emotional development of children in the intervention group was lower than the control group but without significant difference (p＞0.05). However, the rate of suspected social-emotional delay in the intervention group was much higher than the control group (intervention: 29.1%; control:19.9%), illustrating that the Care Group intervention improved the social-emotional development of children who had severe problems.

Table S2 Prevalence of social-emotional delay among intervention and control groups

	Intervention group (n=117)			Control group (n=141)			*χ2*	P value
	Normal	Monitoring	Suspected delay	Normal	Monitoring	Suspected delay		
Baseline	44(37.9%)	23(19.8%)	49(42.2%)	53(38.1%)	42(30.2%)	44(31.7%)	4.621	0.099
Endline	72(61.5%)	30(25.6%)	15(12.8%)	93(66.0%)	32(22.7%)	16(11.3%)	0.542	0.763
Difference	28(23.9%)	7(6.0%)	-34(-29.1%)	40(28.4%)	-20(-8.5%)	-18(-19.9%)	7.713	0.091

a. *$p < 0.05$; **$p < 0.01$

Online Supplementary Document 4: The details of the collection of cost items in the group-based intervention project (in 2019, USD)

4.1 Recruitment costs

Recruitment for our project is divided into two phases. In the first stage, the project team, which mainly included national and county-level experts in maternal and child health care projects, was responsible for recruiting parenting instructors and supervisors. Four supervisors and nine parenting instructors were recruited. The recruitment method is the on-site interview. In the second stage, parenting instructors and supervisors were responsible for recruiting children's parents and children, with 4 supervisors and 9 parenting instructors in charge. The recruitment includes 322 parents of children. Recruitment channels include phone calls, home visits, WeChat, and other new media. In July 2019, the health economics researchers of the project team interviewed a total of 15 staff members on-site, including 9 parenting instructors, 4 supervisors, 1 expert from county-level maternal and child health care center, and 1 national expert. The interview's content includes the interviewees' working situation (whether there is a job, average salary, effective working hours per day), the recruitment way of the interviewees, the recruitment time, and the cost of transportation. All interview data were timely sorted out in Microsoft Excel 2019 (Table S3-1).

The recruitment cost is mainly calculated according to the human capital method, which is divided into two parts: the first is the recruitment time cost, which is calculated according to the hourly wage and recruitment time of the recruitment target; the second is the transportation cost caused by recruitment. The specific calculation formula is as follows:

Recruitment cost = unit time (hour) × unit wage (USD) + transportation cost

Table S3-1 The summary details of recruitment cost

Participant	Average monthly salary (USD)	Average salary per hour	Average Duration of recruitment (minutes)	Transportation cost (USD)	Recruitment cost (USD)
National expert	3891.0	168	41.5	57.9	276.8
County-level expert	506.7	15.2	41.5	57.9	77.7
Supervisor 1	722.1	23.09	30	0.0	107.0
Supervisor 2	144.8	5.68	2.5	0.0	2.2
Supervisor 3	241.3	7.44	0	0.0	0#
Supervisor 4	361.9	12.02	0	0.0	0#
Instructor 1	289.5	7.14	5.5	0.0	6.1
Instructor 2	0*	0	4.5	0.0	0.0
Instructor 3	241.3	12.62	3	0.0	5.8
Instructor 4	160.8	7.72	4.5	0.0	5.4
Instructor 5	723.8	22.72	14.75	0.0	51.7
Instructor 6	0*	0	13.75	0.0	0.0
Instructor 7	144.8	4.55	5	0.0	3.5
Instructor 8	144.8	9.09	17	0.0	23.9
Instructor 9	0*	0	0	0.0	0.0
Total					558.9

Note: * means no job; # indicates no participation in recruitment

According to the calculation of the interview data, the total recruitment cost of this project is USD 558.9, among which the cost of project experts to recruit nurturing instructors and supervisors accounts for the largest proportion, accounting for 63.3% of the total cost.

4.2 Details of training costs

From May 31, 2019 to June 14, 2019, the project team organized a two-stage face-to-face training in Fenxi County. The first phase of the training lasted five days. The second phase lasted four days for a total of nine days. 5 experts, 4 supervisors and 9 nurturing instructors participated in the training phase. The project team supported all the training expenses (including accommodation, transportation, office expenses, and expert labor costs). The health economics researchers of the project team collected the experts, facilitators', and supervisors' time, transportation, accommodation, and office expenses of the whole project through expert interviews and questionnaires. See Table S3-2 for details and costs of training programs.

Table S3-2 The specific cost items of training

Item	Participant	Number	Cost item	Mean
Training (Including two stages)	National and county-level experts	5	Training time per day (hour)	8.0
			Training days	9.0
			Transportation cost (USD)	143.5
			County transportation cost per person (USD)	8.0
			Accommodation fee per day (USD)	13.0
			Food fee per day (USD)	8.7
			Training fee per day (USD)	115.8
			Normal working time per day (hours)	9.6
			Working days per week	5.4
			Average daily wage per hour (USD)	13.7
	Instructors	9	Training time per day (hours)	8.0
			Training days	9.0
			County transportation cost per person (USD)	8.0
			Average hours for travel and training	2.5
			Allowance for transportation and food per day (USD)	21.7
			Normal working hours per day	5.3
			Normal working days per month	15.3
			Average daily wage per hour	7.1
	Supervisors	4	Training time (hour)	8.0
			Training days (day)	9.0
			County transportation cost per person (USD)	8.0

	Average hours for travel and training	2.0
	Allowance for transportation and food per day (USD)	21.7
	Normal working hours per day	8.0
	Normal working days per month	25.8
	Average daily wage per hour (USD)	1.8
Other costs	Material printing fee (USD)	361.9
	Office fee (USD)	464.0

Training costs mainly include the time cost of experts, supervisors and nurturing instructors, transportation cost, accommodation cost, catering cost and office materials cost. Among them, the time cost is calculated by human capital method. The specific calculation formula is as follows:

Training cost = unit time × unit salary (USD) + transportation + accommodation + food and beverage + office expenses

According to the research results, the total training cost of this program is USD 11011.7, among which the time cost of expert's accounts for the largest proportion (50%), which is USD 5507.7.

4.3 Details of material costs

The material cost is divided into two parts. The first part is the costs of material purchased by UNICEF, which mainly includes record cards, hand puppets, toys, colorful crayons, children's books, and USB flash drives. The second part is the supplementary materials purchased by the Fenxi County project team, mainly the Fenxi County project team, including brushes, building blocks, blackboard writing, solid glue, etc. See Table S3-3 for specific material costs.

Table S3-3 The details of material costs

Department	Item	Unit Price (USD)	Number	Service time (Years)	Total price after depreciation (USD)
UNICEF	MP3 player	44.9	10	1	448.8
	USB disk	3.3	10	2	16.6
	Child record card	0.0	100	1	2.9
	Parent Record card	0.0	100	1	4.3
	Children reader.	6.3	196	2	612.9
	Children's learning cards	3.6	50	1	182.4
	Hand puppets	10.2	50	1	512.4
	The doll	26.1	10	2	130.3
	Sand hammer	0.3	60	1	15.6
	Wooden jigsaw puzzle	2.8	50	1	139.0
	A ball	1.1	100	0.5	111.5
	A big ball	0.9	30	0.5	26.9

	Sets of cup	1.6	50	2	39.1
	The mirror	3.0	40	1	121.6
	24 color crayon	1.6	20	0.5	31.3
	A locker	5.8	10	3	19.3
		0.0			0.0
Fenxi County project team	The brush	1.7	6	0.5	10.1
	Building blocks	5.1	11	1	56.3
	Solid glue	0.4	51	0.5	22.1
	Block 2	8.2	12	1	98.1
	scissors	0.5	55	2	13.9
	Paint suit	4.9	10	1	49.1
	Cardboard book	2.1	10	1	21.4
Total (USD)					2686.0

According to the actual service life of materials, the depreciation method is adopted for materials with a service time of more than one year, such as USB disk, children's books, dolls and so on. According to the research results, the total material cost of this project is USD 2686.0, among which the material cost purchased by UNICEF and Fenxi County project team is USD 2414.9 and USD 271.1 respectively.

4.4 Monthly Care Group costs

The intervention frequency of this project was 2 times per month, each activity lasted about 50 minutes, and the intervention was planned for 12 months in total, including July to December 2019, during which monthly intervention activities were normally carried out. From January to June 2020, the activities of the nurturing care group were suspended for the six months due to the COVID-19 outbreak in the country. Parents of children received monthly training from 9 trained parenting instructors, and 4 supervisors conducted monthly supervision, intensive parenting instructor training and home visits. The cost of monthly intervention includes the time cost of preparing and organizing group activities, the time cost of monthly intensive training, the time cost of filling out statements, the time cost of answering WeChat group questions, the time cost of feedback questions, and the time cost of transportation, etc. The health economics researchers of the project team collected the time costs of parenting instructors and supervisors through monthly questionnaires. According to the human capital method, the calculation formula is:

Monthly project intervention cost = unit time spent by parenting instructors (hours) × unit salary of parenting instructors (USD) + Unit time spent by supervisors (hours) × unit salary of supervisors (USD)

The total time cost for the six months from July 2019 to December 2019 is USD 2834.2. From January to June 2020, due to the suspension of group activities, the time cost for these six months is USD 0. The specific calculation results are shown in Table S3-4.

Table S3-4 Monthly intervention cost based on the human time cost approach

Cost item	Time	Cost (USD)
Monthly intervention cost	July 2019	640.3
	August 2019	451.1

	September 2019	493.8
	October 2019	448.4
	November 2019	432.9
	December 2019	367.7
	January 2020-June 2020	0.0
Total (USD)		2834.2

4.5 The quality control of economic data

In the early and middle stages of project implementation, health economic evaluators have been to the project implementation site to conduct cost collection supervision and verification. Provide training and Q&A for cost collection leaders of each project implementation center at the project implementation site to ensure the accuracy of cost collection in the whole cost collection process. During the implementation of the project, each project supervisor was responsible for the cost accounting form of the intervention center in the region, which was filled out once a month. Then the data was input into the Excel form and sent to the health economics evaluation staff by email. Besides, the original input data proof materials were shipped together in the form of photos.

Online Supplementary Document 5: Changes in family nurturing behavior of intervention and control groups

We utilized the early childhood development questions in the Multiple Indicator Cluster Surveys (MICS-6) to assess nurturing behavior. If any household member aged 15 and above engaged in the activity specified, the question was scored "1". Otherwise, it was scored "0". The total score was the sum of the six questions. Notable changes in storytelling and naming/counting/drawing were seen in the intervention group, and the total scores among the intervention group were significantly higher than that of the control group.

Table S4 Changes in family nurturing behavior of intervention and control groups

		Intervention (n=117)	Control (n=141)	p value
Read books or picture books	Pre	0.32(0.47)	0.37(0.48)	
	Post	0.49(0.50)	0.56(0.50)	
	Difference	0.17(0.72)	0.19(0.63)	0.065
Told stories	Pre	0.19(0.39)	0.27(0.45)	
	Post	0.44(0.50)	0.33(0.47)	
	Difference	0.26(0.60)	0.06(0.55)	0.001**
Sang songs	Pre	0.60(0.49)	0.68(0.47)	
	Post	0.67(0.47)	0.69(0.47)	
	Difference	0.07(0.63)	0.07(0.65)	0.900
Took the baby outside the home	Pre	0.61(0.49)	0.72(0.45)	
	Post	0.83(0.38)	0.90(0.30)	
	Difference	0.22(0.54)	0.18(0.48)	0.054
Played with the baby	Pre	0.63(0.48)	0.63(0.48)	
	Post	0.89(0.32)	0.93(0.26)	
	Difference	0.26(0.56)	0.30(0.54)	0.824
Named, counted or drew for/with the baby	Pre	0.38(0.49)	0.50(0.50)	
	Post	0.74(0.44)	0.60(0.49)	
	Difference	0.36(0.58)	0.10(0.69)	0.004*
Total score	Pre	2.71(1.51)	3.17(1.49)	
	Post	4.05(1.32)	4.01(1.40)	
	Difference	1.33(1.75)	0.84(1.72)	0.023*

Online Supplementary Document 6: List of items in the Care Group Toolkit

Table S5 List of items in the Care Group Toolkit

No.	Material	Quantity
1	Baby lotion	1
2	Doll	1
3	Baby blanket	1
4	Breast model	1
5	Baby towel	10
6	Finger puppet	10
7	Sand hammer, rattler, rattle toys	10
8	Colored ocean ball	50
9	Colored blocks	100
10	Disposable plates, small bowls, chopsticks, and	20 pieces each
11	Stainless steel bowl (250 ml)	1
12	Learning cards (animals, fruits, vegetables)	1 set
13	Books	120
14	Ying Yang Bao	10
15	Mirror	10
16	Ball	10
17	Bubble toy	5
18	Empty small box (plastic or papery)	3 (prepared by [illegible]
19	Scotch tape	2
20	Double-sided adhesive tape	2
21	Color paper (A4)	100
22	A4 paper printed with plum tree trunks	10
23	Color paint	1 set
24	Crayon	2 sets
25	A4 paper	100
26	String beads	5 sets
27	Cup toys	5 sets
28	White paper (1m*1m)	1
29	Animal model toys	1 set
30	Plasticine	1 set
31	Adult towel	10
32	Paint brush	2
33	Large cardboard box (the size of a child)	2 (prepared by [illegible]
34	Children scissors	2
35	Glue stick	10
36	Children's songs and music	20-30
37	Storage box	2

Note: The materials are arranged according to the sequence of the *Care Group Manual*
